# What Becomes of the Human Touch in the Age of Generative Artificial Intelligence?

**DOI:** 10.1016/j.mcpdig.2025.100226

**Published:** 2025-05-14

**Authors:** Kishwen Kanna Yoga Ratnam

**Affiliations:** Centre for Non-Communicable Diseases Research, Institute for Public Health, National Institutes of Health, Ministry of Health Malaysia, Setia Alam, Shah Alam, Malaysia

To the Editor:

We are entering an era where generative artificial intelligence no longer simply assists, but begins to create on our behalf. It can craft narratives, shape arguments, and even mimic the tone of human reflection. With this shift, our relationship to knowledge and expression begins to change. Where once we valued the time and thought poured into a piece of writing, we now marvel at how effortlessly it can be assembled. The boundaries between effort and outcome, between process and product, are slowly dissolving. And in that dissolution, a question begins to emerge: what do we truly value in the things we create and in the way we connect?

Consider this very write-up. Does it matter how it came to be, whether shaped by quiet contemplation over many evenings or assembled in moments with the help of a large language model? And do people even care? What once took weeks of introspection can now be produced with a well-phrased prompt.

In this shift, I find myself wondering, how do we now value novelty, effort, and authenticity? Will it matter if something was born of discipline and reflection or simply generated into existence? We may be entering an era where the old saying, "Don’t tell me about the labor, just show me the baby," becomes strangely prophetic.

And yet, something ironic is happening. In a time when artificial intelligence tools can perfect tone and form, I sometimes feel compelled to do the opposite. I might drop in a dash of colloquialism or allow a slight imperfection, not to be careless, but to feel and appear human. I imagine that in the near future, as automation becomes embedded in our digital communications, such as emails, messages, and even status updates, we will all start doing the same. We may begin adding small human touches and deliberate blemishes so that things feel real, a little less polished, but a little more personal.

Speaking of personal, it brings to mind a custom from my Indian Sri Lankan heritage. In our culture, delivering wedding invitations by hand was once a meaningful and respected act. The transition to e-cards was initially met with resistance. But now, even handwritten invitations, which were once deeply cherished, are often relegated to cluttered drawers. Norms evolve not only through disruption but also through subtle adaptation.

So, what becomes the new currency in a world where content is generated and delivery is frictionless? As cliché as it may sound, I believe it is the thought that counts. But not just any thought. It is intention, originality, and authenticity. In a world where fluency can be faked, what becomes rare is not technical mastery, but sincere intention and originality. And in their scarcity lies their value. These qualities cannot be summoned by code or reproduced through prediction. Not truly. Not yet.

Perhaps, in the rhythm of this new world, we will come to see that it is not the ease or elegance of a sentence that matters most, but the depth of intention behind it. Originality, thoughtfulness, sincerity, and authenticity may become the rare elements we look for. Not as relics of a past era, but as signs that something was crafted with care. They are glimpses of the person behind the words. And as we drift further into a future shaped by algorithms, we may find ourselves reaching for what feels imperfect, unpredictable, and real. Not despite its flaws, but because it carries the warmth of something with a soul.

## Potential Competing Interests

The author reports no competing interests.

